# The role and mechanism of vascular wall cell ion channels in vascular fibrosis remodeling

**DOI:** 10.1080/19336950.2024.2418128

**Published:** 2024-10-19

**Authors:** Xiaolin Zhang, Hai Tian, Cheng Xie, Yan Yang, Pengyun Li, Jun Cheng

**Affiliations:** Key Lab of Medical Electrophysiology of Ministry of Education and Medical Electrophysiological Key Lab of Sichuan Province, Collaborative Innovation Center for Prevention and Treatment of Cardiovascular Disease, Institute of Cardiovascular Research, Public Center of Experimental Technology, Hemodynamics and Medical Engineering Combination Key Laboratory of Luzhou, Southwest Medical University, Luzhou, China

**Keywords:** Vascular wall, fibrosis, vascular remodeling, ion channel

## Abstract

Fibrosis is usually the final pathological state of many chronic inflammatory diseases and may lead to organ malfunction. Excessive deposition of extracellular matrix (ECM) molecules is a characteristic of most fibrotic tissues. The blood vessel wall contains three layers of membrane structure, including the intima, which is composed of endothelial cells; the media, which is composed of smooth muscle cells; and the adventitia, which is formed by the interaction of connective tissue and fibroblasts. The occurrence and progression of vascular remodeling are closely associated with cardiovascular diseases, and vascular remodeling can alter the original structure and function of the blood vessel. Dysregulation of the composition of the extracellular matrix in blood vessels leads to the continuous advancement of vascular stiffening and fibrosis. Vascular fibrosis reaction leads to excessive deposition of the extracellular matrix in the vascular adventitia, reduces vessel compliance, and ultimately alters key aspects of vascular biomechanics. The pathogenesis of fibrosis in the vasculature and strategies for its reversal have become interesting and important challenges. Ion channels are widely expressed in the cardiovascular system; they regulate blood pressure, maintain cardiovascular function homeostasis, and play important roles in ion transport, cell differentiation, proliferation. In blood vessels, different types of ion channels in fibroblasts, smooth muscle cells and endothelial cells may be relevant mediators of the development of fibrosis in organs or tissues. This review discusses the known roles of ion channels in vascular fibrosis remodeling and discusses potential therapeutic targets for regulating remodeling and repair after vascular injury.

## Introduction

The main pathological changes that characterize fibrosis are the excessive accumulation of fibrous connective tissue and a reduction in parenchymal cells within the tissues of vital organs, such as the heart, liver, lungs, and kidneys. Sustained progression of fibrosis may impair or abolish organ function, leading to organ failure and mortality, which poses a serious threat to human health and life. Fibrosis can also occur in blood vessels, in addition to the above organs. Vascular fibrosis remodeling usually occurs during the progression of diseases, such as hypertension and diabetes. Collagen, fibronectin, and other extracellular matrix components in the arterial wall accumulate, causing the arterial lumen diameter to shrink and the arterial wall thickness to increase, which affects arterial mechanical function and produces abnormal arterial biomechanics. These changes also increase the risk of developing vascular diseases such as atherosclerosis, arteriosclerosis, aneurysms, and dissection [1,2]. Early treatments that can inhibit the continued progression of fibrosis constitute an important turning point in the treatment of these diseases. Therefore, exploring the potential pathogenic mechanisms that underlie vascular fibrosis and understanding how to reverse fibrosis are timely and clinical problems that need to be addressed.

Ion channels are hydrophilic transmembrane proteins on the plasma membrane that not only play a key role in regulating the physiological functions of excitable cells such as nerve tissue and muscle but also express various types of ion channels in nonexcitable cells. Ion channels also serve as potential targets for regulating the proliferation and differentiation of many cell types. However, ion channel dysfunction or disorder can lead to a variety of diseases, and they are recognized as one of the most crucial classes of drug targets, which have consistently been a focal point of research [3]. Different types of ion channels in fibroblasts, smooth muscle cells and endothelial cells are closely related to the progress of vascular remodeling and fibrosis, and targeted therapy for ion channels may be beneficial for inhibiting persistent fibrotic reactions. In this paper, the possible roles of ion channels in vascular wall smooth muscle cells, endothelial cells, and fibroblasts in the remodeling of vascular fibrosis are summarized. Owing to limited space, the relevant mechanisms of TRP, KCa and vascular fibrosis remodeling have been described.

## Physiological function of ion channels on vascular cells in the vascular system and them roles in vascular diseases

There are a variety of distinct ion channels in the human vascular system. These ion channels play important roles in the physiological regulation of the cardiovascular system, including regulating the membrane potential, mediating signal transduction, and altering hemodynamics and vasomotor function. Ion channels can also participate in pathophysiological reactions during disease states and play an important role in maintaining vascular homeostasis [4,5].

Various types of functional ion channels are expressed on arterial endothelial cells. These include calcium-activated potassium channels (SK, IK), inward rectified K^+^ channels (Kir), ATP-sensitive K^+^ channels (K-ATP), voltage-gated K^+^ channels (Kv), Ca^2+^-activated Cl^−^ channels (CaCCs), and TRP channels [6]. The channels that regulate calcium influx in endothelial cells include calcium-activated potassium channels, voltage-dependent calcium channels, and TRP channels, which are activated in response to changes in the physical and chemical environment, resulting in Ca2+ influx into cells via a variety of pathways.

Calcium-mediated signal transduction within endothelial cells involves the release of various factors, including NO, prostacyclin (PGI2), and platelet-activating factor (PAF), which further participate in normal physiological responses or pathological reactions of the vasculature [7]. By combining single-cell microfluorimetry with perforated patch clamp recordings, Braun and colleagues [8] reported that in human umbilical vein endothelial cells (HUVECs), stimulation with agonists (ATP and histamine) led to cytoplasmic Ca^2+^ transients and membrane hyperpolarization in HUVECs and directly induced nitric oxide synthesis in human vascular endothelial cells. Mechanistically, under the stimulation of humoral factors or hemodynamic force, intracellular calcium in ECs increases and activates SK and IK on ECs, mediating endothelial hyperpolarization. This hyperpolarization provides an electrochemical driving force for Ca^2+^ influx and activates endothelial NO synthase by regulating Ca^2+^ inflow. It promotes the dependent synthesis of Ca^2+^-vasodilator factors (NO, epinephrine, and EDHF). In addition, endothelial cells also make direct contact with VSMCs via the myoendothelial space junction (MEGJ) and conduct electrical current, causing hyperpolarization of adjacent smooth muscle and subsequent vasodilation [9].

The ion channels expressed on the vascular smooth muscle in the medial artery include inward rectified K^+^ channels, calcium-activated K^+^ channels, voltage-dependent K^+^ channels, voltage-dependent Ca^2+^ channels, TRP channels, etc. Calcium-activated K^+^ channels, voltage-related Ca^2+^ channels and TRP channels on smooth muscle cells work together to maintain the homeostasis of the intracellular Ca^2+^ concentration, and Ca^2+^ ions help maintain the electrochemical gradient in cells to ensure the normal excitability of cells [10]. The control of Ca^2+^ entry by these ion channels also determines the contractile function of SMCs and regulates vascular tone.

The arterial adventitia contains several cell types, including fibroblasts, immune regulatory cells (dendritic cells and macrophages), progenitor cells, and pericytes, and plays a crucial role in regulating vessel function and structure. For example, perivascular cells undergo alterations in their functional and phenotypic characteristics in response to vascular stress or injury, thereby fostering the development of chronic vascular inflammation that ultimately impacts the tension and structure of the vessel wall [11].

Under pathological conditions, ion channels in the vascular system may exhibit aberrant expression and distribution, resulting in dysfunctional behavior that exacerbates vascular damage, such as disruption of vascular permeability and promotion of pathological remodeling of the vasculature [12]. For example, studies have shown that transient receptor potential 3 (TRP3) channels are associated with the pathogenesis of hypertension. The TRPC3 channel in vascular smooth muscle cells and aortic tissue is upregulated in hypertensive animal models [13] as well as in hypertensive patients [14]. Another study revealed that the serine/threonine kinase WNK4 regulates TRPC3 activity and promotes vascular smooth muscle cell (VSMC) hypertrophy, which promotes myogenic tension and exacerbates hypertension [15]. Transient receptor potential 4 (TRPV4) channels expressed on arterial adventitia fibroblasts play important roles in the development of adventitia remodeling induced by pulmonary hypertension [16].

## Ion channel and vascular fibrosis remodeling

**Figure 1.**
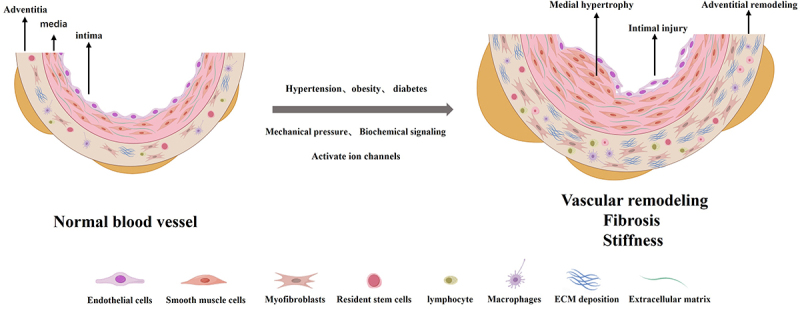
The known pathways by which abnormal activation of ion channels can promote the development of vascular remodeling.

The occurrence and progression of many diseases are accompanied by pathological abnormalities in vascular fibrosis remodeling, including the systemic vascular remodeling and fibrosis induced by obesity through inflammation or oxidative stress associated with hyperglycemia [17]. Arteritis (TAK), a chronic large vessel disease, is characterized by thickening of aortic fibrosis, arterial narrowing, and microstructural distortion [18]. In addition, several studies have reported that Ang-II-induced hypertensive vascular remodeling leads to excessive deposition of extracellular matrix collagen in the adventitia, increased fibrosis, and decreased arterial compliance [19]. The key process of vascular fibrosis remodeling is that vascular cells, including smooth muscle cells, fibroblasts, and endothelial cells, perceive mechanical or chemical stimuli in the surrounding environment and convert them into biochemical signals in various ways to produce biological effects such as cell hypertrophy and proliferation, as well as increased protein synthesis or increased extracellular matrix deposition. The possible pathways involved in the remodeling process of vascular fibrosis include signal transduction involving ion channels on vascular cells (smooth muscle cells, endothelial cells, fibroblasts, etc.), activation of various tyrosine kinases, and autocrine production and release of growth factors [20]. Different types of ion channels on vascular cells, including endothelial cells, smooth muscle cells and fibroblasts, play different roles in the process of vascular remodeling and fibrosis. Different types of ion channels, such as TRP, Kca, mechanosensitive ion channels, and volume-regulated anion channels, are involved in the process of fibrosis in different organs or tissues. The following section focuses on the relationships between these classes of ion channels and vascular fibrosis. As depicted in Figure 1: Causes of vascular remodeling and fibrosis.

### Transient receptor potential (TRP) channels and vascular fibrosis remodeling

In humans, the TRP superfamily consists of 28 subtypes, including six subfamilies: TRPC1–7, TRPV1–6, TRPM1–8, TRPA1, TRPML1–3, and TRPP1–2 [21]. Most TRP ion channels are permeable to Ca^2+^ with the exception of TRPM4 and TRPM5. Therefore, TRP channels constitute an important pathway for Ca^2+^ to enter the cell [22]. Members of the transient receptor potential (TRP) protein superfamily act as sensors for detecting and transducing the physical and chemical state of the intracellular microenvironment, and they are activated in response to thermal, chemical, and mechanical stimuli [23]. The regulation of Ca^2+^-TRP ion channels involves three main mechanisms: Ca^2+^ binds directly to the vicinity of the TRP domain and to two negatively charged residues in the TRP domain to reduce the sensitivity of TRP to Ca^2+^ [24]; Ca^2+^ binding with calmodulin (CaM) induces structural changes in TRP channels or activates downstream kinases and phosphatases, regulates self-phosphorylation of TRP channels and controls channel activation and deactivation. Intracellular Ca^2+^ activates Ca^2+^-sensitive PLC and induces cleavage of PIP_2_ [25]. Studies have reported that several TRP channels may play central roles in the progression and prevention of fibrotic proliferative diseases in the lung, heart, liver, kidney, brain, blood vessels and other important internal organs [21]. Several TRP channels related to vascular remodeling and fibrosis are briefly described below.

#### TRPM7 and vascular fibrosis remodeling

As a magnesium transporter in the transient receptor potential melastatin (TRPM) cation channel family with channel and kinase domains, TRPM7 is universally expressed throughout the body [26]. TRPM7 is a permeable channel for bivalent cations such as Ca^2+^, Zn^2+^ and Mg^2+^ [27,28]. TRPM7 regulates many cellular processes, including Mg^2+^ homeostasis, cell growth/apoptosis, and differentiation [29,30]. Previous studies have shown that Mg^2+^ is involved in many basic physiological and biochemical processes that can regulate cardiovascular function, including vasoconstriction and dilation, vascular inflammation and protein synthesis [31,32]. Magnesium is also a key cation in most enzymatic reactions and is essential for proper cardiovascular function. Mg^2+^ deficiency can lead to endothelial dysfunction, arterial remodeling, and hypertension, and normalization of Mg^2+^ improves vascular function and lowers blood pressure [27]. On the other hand, Mg^2+^ extensively regulates growth processes associated with remodeling and fibrosis, and at the subcellular level, these effects occur at least in part through Mg^2+^-dependent regulation of mitogen-activated protein (MAP) kinases, tyrosine kinases and reactive oxygen species, which are important signaling molecules involved in proliferation, fibrosis, and inflammation [33,34]. Owing to the ability of TRPM7 to penetrate Mg^2+^, abnormal TRPM7 expression/activity may lead to impaired intracellular free Mg^2+^ concentrations, VSMC systolic dysfunction, abnormal cell proliferation, inflammation, and fibrosis [35]. For example, HEK-293 cells deficient in the TRPM7α-kinase domain secrete the proinflammatory mediators ROS, ICAM-1, Cox-2, and PAI-1 in response to aldosterone stimulation, resulting in proinflammatory phenotypes [36]; TRPM7 kinase-deficient heterozygous mice with Ang II infusion present increased blood pressure, vascular dysfunction, cardiovascular inflammation and fibrosis [37]; and the expression of proinflammatory factors, such as Ang II and reactive oxygen species (ROS), increases TRPM7 expression in VSMCs [38]. Vascular wall thickening is induced by pressure overload in transverse aortic contraction (TAC) rats, the accumulation of exogenous vascular collagen, and increased TRPM7 levels in vascular outer membrane fibroblasts [39]. TRPM7 is abnormally expressed and distributed under pathological conditions; thus, targeting TRPM7 can play a vascular protective role, which has been verified in animal models in which TRPM7 is knocked out or inhibited. In the vascular system, inhibition of TRPM7 reduces calcification in VSMCs induced by high inorganic phosphates [40]; disruption of platelet TRPM7 kinase activity protects mice from arterial thrombosis and ischemic stroke [41]. In addition, Liu J et al [42] reported that mechanosensitive TRPM7 converts mechanical signals into biological signals, activates TRPM7 under mechanical tensile stress (MSS), promotes the transformation of vascular outer membrane fibroblasts into myofibroblasts through the p38 MAPK/JNK pathway, promotes inflammation and finally mediates vascular fibrosis.

#### TRPC6 in fibroblasts, smooth muscle cells, and endothelial cells mediates vascular fibrosis

Transient receptor potential 6 (TRPC6) is a nonselective cation channel that permeates Na+ and Ca^2+^. The TRPC6 channel is directly activated by the second messenger diglycerol (DAG), and the TRPC6 protein is also regulated by specific tyrosine or serine phosphorylation and phosphoinositol. The binding of intracellular Ca^2+^ with calmodulin enhances channel activity [43]. As a calcium ion channel, TRPC6 regulates calcium-operated calcium entry (SOCE) [44]. Calcium store-operated intracellular calcium entry (SOCE) is the main pathway of intracellular calcium flow and the main mechanism of regulating intracellular calcium homeostasis and cell function [45]. The channels for storage manipulation include Ca^2+^-selective CRAC channels involving Orai 1 and cation-permeable SOC channels formed by Orai and TRPC subunits [46], which are the main ion channels that regulate SOCE. Under pathological conditions such as pulmonary hypertension, changes in TRPC6 expression and function affect SOCE. For example, Masson et al [47,48] reported that the protein expression of TRPC6 in the pulmonary artery smooth muscle (PAH-hPASMCs) of patients with pulmonary hypertension was increased, and the SOCE of PAH-hPASMCs was increased twofold, accompanied by increased calcineurin activity, compared with that of hPASMCs in the control group. TRPC6 knockdown in PAH-hPASMCs reduced SOCE by 30%. Davis et al [49] reported that TRPC6 is a major mediator of TGFβ-dependent SOCE enhancement in activated fibroblasts and that the overexpression of TRPC6 in fibroblasts and the use of exogenous TGFβ and Ang II significantly increased SOCE signaling in fibroblasts, which was not observed in Trpc6^−/−^ fibroblasts. In addition, p38 MAPK inhibitors significantly reduced the TGF-β-dependent increase in SOCE in fibroblasts, suggesting that the p38/TGF-β signaling pathway relies on Trpc6 and downstream Ca^2+^ signaling to enhance fibroblast transformation. TGF-β and angiotensin II activate the p38 mitogen-activated protein kinase (MAPK) serum response factor (SRF) signaling pathway or act on the TRPC6 promoter to induce TRPC6 expression during fibroblast differentiation into myoblasts. However, increased SOCE resulting from increased TRPC6 expression provides sufficient Ca^2+^ to activate calcineurin – NFAT signaling to promote fibroblast differentiation. Jain et al [50,51] reported the upregulation of TRPC6 channels in PASMCs from PAH patients. Elevated cytoplasmic Ca^2+^ concentrations in PASMCs lead to PASMC contraction, sustained pulmonary contraction and pulmonary remodeling. An increased Ca^2+^ concentration in VSMCs triggers the activation of the Ca^2+^-PI3K/AKT/mTOR pathway, and the dedifferentiation of VSMCs from a systolic or resting phenotype to a proliferative or synthetic phenotype leads to excessive pulmonary vascular remodeling [52]. In a mouse model of pulmonary hypertension with significant remodeling and fibrosis of the outer and media membranes of the large and small pulmonary arteries along with a complex biological process called endothelial mesenchymal transformation (EndMT), inhibiting the activation of the TRPC6/calcineurin/NFAT signaling pathway can inhibit the EndMT process in pulmonary artery endothelial cells, thereby delaying or preventing the occurrence of PAH and pulmonary vasoconstriction and excessive remodeling, which also indicates the role of TRPC6 as a potential therapeutic target to inhibit vascular remodeling and fibrosis in pulmonary hypertension [51,53].

#### TRPV4 and vascular fibrosis

##### Basic structure and physiological functions of TRPV4

The TRPV4 channel consists of four monomers, and each monomer of TRPV4 consists of six transmembrane domains, with a ring of pores between the fifth and sixth transmembrane domains. The N-terminus and C-terminus are located in intracellular regions and contain a variety of functional domains, such as the proline-rich N-terminus and the 6 ankyrin domains of TRP, the C-terminal calmodulin domain, the ankyrin domain and the proline-rich domain, which interact with other proteins to regulate downstream signaling processes [54]. TRPV4 penetrates mainly Ca^2+^ and Mg^2+^ nonselective cation channels, which can be activated by physical and chemical stimuli, including temperatures near 27°C, osmotic pressure changes, mechanical stress and pH changes, and can also be activated by interactions with endogenous molecules, such as arachidonic acid (AA) and its derivatives, PIP_2_, etc [55]. Kwon et al [56] analyzed the structure of TRPV4 via cryoelectron microscopy. RhoA is the main Rho GTPase that binds to TRPV4; TRPV4 channel activation is related to rigid rotation of the ankyrin repeat domain in the cell, and the interaction of TRPV4 with membrane-attached RhoA limits this rotation and regulates channel activity. Understanding the structural basis of ligand-dependent TRPV4 gating and the channel regulation of RhoA will enhance drug design for TRPV4-dependent diseases. For example, the administration of TRPV4 antagonists improves negative outcomes in animal models of pulmonary edema, blood‒retinal and blood‒brain barrier disruption, and peripheral neuropathy [57,58], and treatment with the TRPV4-specific antagonist AB159908 reduces Ca^2+^ inflow into fibroblasts, blocks collagen remodeling, and maintains the balance of ECM components [59].

TRPV4 function has been well established in endothelial cells via calcium influx and patch clamp assays, but its functional expression in fibroblasts is still under investigation. TRPV4 expressed in endothelial cells is activated by vascular stretching and shear stress stimulation, and TRPV4-mediated calcium flow through PI3 kinase activates additional integrins, thereby inducing instantaneous downregulation and subsequent stabilization of Rho, promoting cytoskeletal reorientation and directing the migration of ECs required for physiological angiogenesis. In addition, TRPV4-mediated calcium influx can activate endothelial nitric oxide synthase (eNOS) and produce nitric oxide (NO), thereby inducing vasodilation [60].

##### TRPV4 in fibroblasts with vascular fibrosis

During the development of tissue and organ fibrosis, along with ECM remodeling, cells exert a contractile force on matrix polymers and combine the assemblages of collagen fibers into rigid aggregates. The ECM stiffness increases. Because TRPV4 channels can be activated by mechanical force, they can sense changes in ECM stiffness and then mediate actin remodeling. Therefore, TRPV4 May play a role in fibroblast differentiation and fibrosis [61]. Studies of TRPV4 expression and activity in fibroblasts suggest that increased TRPV4 expression is associated with skin fibrosis in scleroderma [62] and that the activity of TRPV4 channels is increased in the lung fibroblasts of patients with idiopathic pulmonary fibrosis [63]. TRPV4 knockout mice are protected from lung, skin, heart, and corneal fibrosis and are spared the negative effects of respiratory remodeling and epangitis [64]. Regarding the profibrotic role of TRPV4 in the vascular system, Cussac et al [16] reported that TRPV4 channel-mediated pulmonary artery fibroblast (PAF) activation in pulmonary hypertension led to excessive outer membrane remodeling characterized by increased connective tissue deposition. It is known that TRPV4 protein is upregulated in the artery adventitia of rats subjected to chronic hypoxia and monocrotaline-induced pulmonary hypertension. In a trpv4^−/−^ PH model, adventitial remodeling was weakened. In vitro studies have suggested that TRPV4 can promote the proliferation and migration of PAFs. The activation of TRPV4 promotes the synthesis of extracellular matrix proteins (type I collagen and fibronectin) [16]. Adapala et al [65] reported that the use of the TRPV4 agonist GSK1016790A promoted TRPV4-mediated calcium influx in cardiac fibroblasts in a dose-dependent manner in the 10–300 nM concentration range. GSK1016790A at 100 nM doubled the calcium inflow in TGF-β1-treated fibroblasts, and the use of the TRPV4 agonist GSK1016790A can upregulate the expression of α-SMA and matrix molecules in fibroblasts, promoting the production of collagen. The composition of the ECM is misaligned, and the ECM is deposited [64]. Administration of TRPV4 antagonists reversed the TGF-β1-induced increase in calcium inflow, and administration of the TRPV4 antagonist AB159908 or TRPV4 knockdown significantly decreased the expression of TGF-β1-induced α-smooth muscle actin (α-SMA). In addition, by preparing gelatin hydrogels with different hardnesses (98–2280 Pa), it was found that the fibroblasts attached to the gel with high stiffness (2280 Pa) had a larger spreading area and high expression of α-SMA. The use of the TRPV4 antagonist AB159908 significantly reduced the increase in cell area and α-SMA expression under these conditions. These findings suggest that the mechanosensitive ion channel TRPV4 promotes myofibroblast activation by integrating mechanical signals from TGF-β1 biochemical signals and ECM components to promote fibrotic function [65]. In summary, TRPV4 plays a unique role in fibroblast activation and matrix remodeling, and the dysregulated ECM remodeling caused by TRPV4 dysfunction is closely related to a variety of cardiovascular diseases and fibrosis.

### Calcium activation of potassium channels and vascular fibrosis remodeling

Ca^2+^ is both an essential bivalent cation in the body and a secondary messenger within numerous signal transduction pathways that regulate various physiological functions of many different cells throughout the body and determining a variety of cell fates, including cell growth, differentiation, and apoptosis [66]. Calcium-activated potassium channels (Kca) play an important role in cellular excitability by sensing and responding to changes in the intracellular Ca^2+^ concentration. According to their conductance, the K_Ca_ family have been divided into small conductance (SK, ~ 4–14 pS), medium conductance (IK, ~ 32–39 pS), and large conductance (BK, ~ 200–300 pS) channels [67]. K_Ca_ is one of the main channels regulating the internal flow of external Ca^2+^. K_Ca_ channels help control the membrane potential and depolarize the membrane through the entry of positively charged calcium. K_Ca_ maintains a negative membrane potential by increasing the intracellular calcium concentration, which helps maintain the entry of calcium into cells, participates in the calcium signaling process, and regulates various cellular activities [68,69]. However, when Ca^2+^ homeostasis is disturbed or maladjusted, an excessive increase in or continuous depletion of intracellular Ca^2+^ leads to a variety of functional abnormalities. Examples include abnormal cell excitability, tumor growth, apoptosis, and many remodeling processes associated with inflammation and, consequently, slow progression and often irreversible fibrosis [70–72].

#### Basic structure and physiological function of BK in smooth muscle cells

The BK channel consists of α and β subunits. The pore-forming BKα subunit plays an important role in the transmembrane function of potassium channels. The auxiliary BKβ1 subunit is a relatively small membrane protein with two transmembrane segments [73]. The BKβ1 subunit is sensitive to changes in the membrane potential and Ca^2+^ concentration of BK channels. In the absence of the BKβ1 subunit, the BK channel expressed in vitro has a significantly reduced susceptibility to Ca^2+^, opening significantly only at more depolarized membrane potentials [74]. BK channels exist in the vasculature and are highly expressed in vascular smooth muscle cells. In VSMCs, locally released spontaneous activating Ca^2+^ or agonist (Ach) stimulates the release of Ca^2+^ in the sarcoplasmic reticulum from the channel through the lanidine receptor [75], causing an increase in the intracellular Ca^2+^ concentration to activate BK channels [76]. Extracellular Ca^2+^ inflow through L-type voltage-gated Ca^2+^ channels (Cav1.2) also stimulates BK channel activity [77]. BK activity contributes significantly to the regulation of vascular function because of its large single-channel conductance (200–300 pS) and high protein expression [78]. The opening of the BK channel can cause the expulsion of K^+^, resulting in hyperpolarization of the membrane. This change in membrane potential shuts off Cav1.2, which in turn reduces Ca^2+^ inflow and induces vasodilation. In contrast, the closure of BK channels causes membrane depolarization, which opens Cav1.2, leading to Ca^2+^ inflow and consequent vasoconstriction [79].

BK channel opening in VSMCs depends on a local increase in Ca^2+^ in the VSMC cytoplasm (Ca^2+^ spark). Brenner et al [76] reported that the Ca^2+^ spark site was very close to the BK channel and that the BK channel was activated when the local Ca^2+^ concentration reached the micromolar level. The importance of calcium sparks is that they help maintain the concentration of calcium ions within cells, triggering muscle contraction of skeletal and cardiac muscles and muscle relaxation of smooth muscles. The appearance of Ca^2+^ sparks almost always temporarily activates a set of BK channels to generate transient outgoing potassium currents (STOCs). Tight coupling of Ca^2+^ sparks and STOCs, interference with Ca^2+^ spark formation or BK channel opening to prevent membrane repolarization lead to VSMC contraction. Thus, BK channel activity in vascular smooth muscle cells (VSMCs) is associated with spontaneous transient outgoing potassium currents (STOCs) [80]. BK channels act as negative feedback modulators of vascular tension by linking membrane depolarization and local increases in the intracellular Ca^2+^ concentration (Ca^2+^ sparks) to repolarize spontaneous transient outgoing potassium currents (STOCs) [81]. BK series channels are involved in various physiological functions, including setting the resting membrane potential in VSMCs, regulating the tension of the vascular bed, and regulating the release of hormones and neurotransmitters [79,82].

#### BK on smooth muscle cells and vascular fibrosis

Abnormal expression or dysfunction of BK channels has been linked to many vascular diseases, as further demonstrated by the targeting of the missing BK subunit in VSMCs. Pluger et al [81] demonstrated that the removal of BKβ1 subunits from the BK channel in VSMCs resulted in decreased channel activity. They recorded STOC of isolated cerebral artery vascular smooth muscle cells using a perforated patch clamp technique. STOC was observed in BKβ1^+/+^ VSMCs with a test potential of −50 mV. However, the occurrence of STOCs in BKβ1 ^−/−^VSMCs shifted to a more positive membrane potential. The STOC frequency of BKβ1^−/−^ VSMCs reached the same level as that of BKβ1^+/+^ cells only when the membrane potential was more depolarized. Compared with that of BKβ1^+/+^ cells, the STOC frequency of BKβ1^−/−^ VSMCs was only 10% of that of BKβ1^+/+^ cells at near-normal resting membrane potential (−40 mV). The Ca^2+^ spark/STOC relationship in BKβ1^−/−^ VSMCs is uncoupled. Loss of the BKβ1 subunit reduces the sensitivity of BK to Ca^2+^, resulting in impaired coupling between Ca^2+^ sparks and BK channel activity. A STOC frequency approaching normal can only be achieved by reaching a more depolarized VSMC membrane potential, which leads to the contraction of VSMCs and increases the systemic blood pressure of transgenic mice. Xu et al [83] have reported that BKβ1 subunit knockout in VSMCs promoted vascular remodeling and fibrosis in obese mice induced by a high-fat diet. The possible mechanism involved an increase in Ca^2+^ in VSMCs combined with hyperglycemia induced by a high-fat diet to inhibit BK channel activity. Moreover, BK channel knockdown stimulates SMC proliferation by disrupting the stability of apoptosis/proliferation in the vascular media layer and ultimately mediates vascular remodeling and fibrosis. The role of BKα subunits in cardiovascular pathology is unclear. Sausbier et al [84] reported that BK α-subunit knockout mice with VSMCs presented a loss of spontaneous transient outward current (STOC) and increased depolarizing membrane potential, resulting in increased systemic blood pressure in BK α-subunit knockout mice. Compared with WT rats, BKα subunit knockout rats presented a smaller inner diameter, thickened aortic wall, increased collagen fiber deposition, disordered elastic fiber arrangement, and increased fibrosis. It is also known that c1q/tumor necrosis factor associated protein 7 (CTRP7), the downstream target gene of the BKα subunit, is expressed in vascular smooth muscle cells (VSMCs). BKα knockdown can reduce the level of CTRP7, activate the PI3K/Akt pathway, promote vascular inflammation, in combination these changes will ultimately lead to the adverse progression of vascular remodeling and fibrosis [85].

The renin‒angiotensin system is a central component of the physiological and pathological responses of the cardiovascular system. The primary effector hormone angiotensin II (ANG II) has direct physiological effects, including vasoconstriction and blood pressure regulation. Angiotensin II induces the cleavage of 4,5-diphosphate inositol (PIP _2_) by binding to the angiotensin II receptor (AT1R), resulting in the delivery of inositol triphosphate (IP 3) and diglycerol (DAG) in vascular smooth muscle cells. Binding of IP 3 to the IP 3 receptor (IP 3 R) induces the release of Ca^2+^ from the sarcoplasmic reticulum into the cytoplasm to increase the intracellular Ca^2+^ concentration and induce vasoconstriction [86]. In addition, Ang II also mediates the activation of NAD(P) H oxidase through AT1 receptors, leading to the production of reactive oxygen species, which are widely associated with vascular inflammation and fibrosis. AT1R regulates BK channels through tight protein‒protein interactions involving multiple BK regions [87], which allows ANG II to directly or indirectly regulate BK channels. Numerous studies have shown that Ang II is heterogeneous in its ability to regulate BK channel activity and that BK is closely related to the remodeling of the vasculature induced by Ang II. To investigate the inhibitory effect of Ang II on BK channels, Toro et al [88] tested whether Ang II affects BK channels at the single-channel level by incorporating BK channels from primary coronary SMCs into the lipid bilayer and reported that Ang II inhibited K+ currents in a dose-dependent manner. Minami et al [89] reported the same observation in porcine coronary artery SMCs. Zhang et al [87] demonstrated that Ang II-induced BK channel current reduction was AT1R dependent after simultaneous transfection of BKα and AT1R in HEK293T cells.

Effect of Ang II on BK channel activation. By using fresh intestinal muscle cells from the ileum of guinea pigs, Romero et al [90] reported that Ang II or its synthetic analogs can increase BK channel activity because their stimulation causes dosing-dependent activation of Ca^2+^ in BK channels in a high-K^+^ solution. The activation effect of Ang II on the BK channel can be eliminated by the specific AT1R blocker losartan [91]. Varo et al [92] reported that long-term treatment with losartan in SHRs reduced the expression of tissue inhibitor of metalloproteinase 1 (TIMP-1), increased collagenase activity, promoted the degradation of collagen fibers, and thus improved myocardial interstitial and perivascular fibrosis. Additionally, Brilla et al [93] reported that the use of the angiotensin-converting enzyme inhibitor lisinopril can enhance the collagen degradation ability of tissue matrix metalloproteinase-1 in SHRs and alleviate the interstitial and perivascular fibrosis of myocardial coronary arteries. On the other hand, Albarwani et al [94] reported that treatment with lisinopril increased the expression of eNOS, SK, and BK channel proteins in SHRs and improved the contribution of NO to ACh-mediated vascular relaxation, but they did not conduct further studies on how lisinopril regulated BK. Considering the functional heterogeneity of BK in response to Ang II, BK channels and Ang II receptors in VSMCs in hypertensive states may be potential targets for the regulation of blood pressure and the treatment of vascular remodeling.

#### Basic structure, physiological function and vascular fibrosis of IK

The medium conductance Ca^2+^ activated K^+^ channel KCa3.1 (also known as KCNN 4) is a calcium-activated intermediate conductance potassium ion channel. KCa3.1 is composed of four KCa3.1 subunits and four calmodulin molecules and is highly sensitive to change in intracellular Ca^2+^. Ca^2+^ controls its activation and inactivation by binding to calmodulin via KCa3.1 and participates in the regulation of the cell membrane potential and intracellular calcium signaling [95]. KCa3.1, which is expressed on vascular endothelial cells, has been shown to play a role in regulating endothelium-dependent vasodilation. Endothelial cells regulate vasodilation by releasing vasoactive factors such as nitric oxide, prostacyclin, and the endothelium-derived hyperpolarization factor EDHF. Ca^2+^-activated K^+^ (KCa) channels mediate endothelial hyperpolarization under humoral or hemodynamic stimulation, and this hyperpolarization provides an electrochemical driving force for Ca^2+^ influx and promotes the dependent synthesis of Ca^2+^-vasodilator factors [96]. In transgenic animals with genetic defects in KCa3.1, EDHF-mediated vasodilation is greatly reduced, and arterial blood pressure is increased [97]. In addition, KCa3.1 plays an important role in regulating cell proliferation, differentiation, migration, mediator release, and wound healing [98,99].

KCa3.1 regulates intracellular calcium signaling, maintains calcium homeostasis, and regulates vascular function, making it critical, and intracellular free Ca^2+^ acts as a ubiquitous secondary messenger that regulates many cellular functions, such as secretion, metabolism, differentiation, proliferation, and contraction. For many of these functions, extracellular Ca^2+^ influx is essential. Acute Ca^2+^ influx stimulated by TGF-β1 induces intracellular Ca^2+^ oscillation, promoting the opening of KCa3.1 channels and thus forming a positive feedback loop. Ca^2+^ influx contributes to the activation of classic fibrotic pathways, such as the Smad2/3, P38 and Ras pathways. Thus, pathological KCa3.1 dysfunction is also linked to many fibrotic diseases, including idiopathic pulmonary and renal tubulointerstitial fibrosis and cardiovascular fibrotic diseases. In cell types such as fibroblasts and myofibroblasts, which play a central role in fibrotic diseases, targeting KCa3.1 and regulating extracellular Ca^2+^ inflow can inhibit most TGF-β1 and growth factor-dependent profibrotic signaling pathways, thus inhibiting or treating fibrotic diseases [100]. Many studies have also shown that pharmacological inhibition and genetic deletion of KCa3.1 in mice can prevent or alleviate fibrosis, including blocking KCa3.1, which alleviates paraquat (PQ)-induced lung inflammation and fibrosis. Blocking KCa3.1 also decreases type I collagen and α-SMA levels and fibroblast proliferation [101]. Pharmacological inhibition or gene deletion of KCa3.1 alleviates diabetic renal interstitial fibrosis in mice by inhibiting fibroblast activation and phosphorylation of Smad2/3 and ERK1/2 activated by TGF-β1 [102]. Blocking KCa3.1 decreased collagen secretion in TGF-β1-dependent fibroblasts [98]. In a corneal fibrosis mouse model induced by ocular alkali burn, KCa3.1 knockout significantly reduced the expression of genes associated with corneal fibrosis, collagen I, α-smooth muscle actin (α-SMA) and other profibrotic genes [103].

In the cardiovascular system, Kca3.1 has also been linked to the development of vascular inflammatory diseases. Zhu et al [104,105] reported that the level of KCa3.1 was increased in the coronary artery vessels of patients with coronary artery disease and that the expression and activity of KCa3.1 in VSMCs and macrophages infiltrating atherosclerotic lesions were increased, which promoted the accumulation of VSMCs and macrophages in plaques and their proliferation and formation. Differential expression of KCa3.1 channels may enable KCa3.1 inhibitor to selectively target diseased tissues, and selective pharmacological blockade and gene silencing inhibit KCa3.1-mediated proliferation, migration, and oxidative stress. PDGF is a mitogen widely used in vitro and in vivo [106]. PDGF stimulation of VSMCs resulted in a time-dependent increase in KCa3.1 mRNA expression, and whole-cell patch-clamp experiments revealed that PDGF induced a significant increase in functional KCa3.1 channels in VSMCs. Applying a ramp voltage of −120 mV to 40 mV and 3 μM free calcium can stimulate the KCa3.1 current in VSMCs, and the amplitude of the KCa3.1 current increases after PDGF stimulation of VSMCs. TRAM-34 (IC50 of 18 nM) can block the KCa3.1 current stimulated by PDGF. Inhibiting channel activity. In addition, in vivo therapy with the KCa3.1 inhibitor TRAM-34 and clotrimazole significantly reduced the development of aortic atherosclerosis in Apoe^−/−^ mice by inhibiting VSMC proliferation and migration to plaques, reducing plaque infiltration by macrophages and T lymphocytes, and reducing oxidative stress [105]. The KCa3.1 channel can be used as a therapeutic target for chronic vascular inflammatory diseases [107].

#### Endothelial SK and vascular fibrosis remodeling

The small conductance Ca^2+^-activated K^+^ channel (SK) is gated by intracellular Ca^2+^, activated as the intracellular calcium level increases, and provides hyperpolarized K^+^ conductance, which is involved in a wide range of physiological processes, including the basis of neuronal excitability, rhythmic hormone release from adenocytes, and smooth muscle tone [108,109]. The SK channel is encoded by three genes, and the members of the SK family include SK1, SK2, and SK3. The KCNN1 gene encodes the human SK1 channel (KCa2.1); the KCNN2 gene encodes the SK2 (KCa2.2) channel; the KCNN3 gene encodes the SK3 (KCa2.3) channel; the SK channel is sensitive to apamin; and the unit conductance value is ~ 4–14 pS [110]. SK is a heteropolymer complex containing a pore-forming alpha subunit and the Ca^2+^-binding protein calmodulin (CaM). When Ca^2+^ binds to the EF hand in the CaM N lobe, the trigger channel opens [111]. SK is a high-affinity calcium sensor that can be activated by submicromolar concentrations of intracellular calcium and translate fluctuations in intracellular calcium concentrations into changes in the membrane potential [112]. In the cardiovascular system, SK is expressed in both endothelial cells and smooth muscle cells [109,113]. During endothelium-dependent vasodilation and blood pressure regulation, the Ca^2+^ concentration in endothelial cells increases and activates SK channels, hyperpolarizes ECs and nearby SMC membranes and causes vasodilation [114].

NO is a major vasodilator molecule, and under normal circumstances, an increase in Ca^2+^ in endothelial cells activates endothelial NO synthase (eNOS). Endothelium-derived NO diffuses to SMCs, increasing cyclic guanosine phosphate (cGMP) levels and cGMP-dependent kinase (PKG) activity, leading to SMC relaxation [115]. eNOS activity and NO-cGMP signal transduction are impaired in PH patients and PH model mice. In PH, endothelial dysfunction and sharply reduced levels of endothelium-derived vasodilators lead to impaired NO-mediated vasodilation [116]. In this case, EDH may serve as a backup system to maintain overall endothelial function, and recent studies have shown that IK channels and SK channels, as therapeutic targets for PH, can reduce vascular remodeling in PAP and PH in an endothelium-dependent manner. In cardiovascular disease (including hypertension, obesity, diabetes, and atherosclerosis), SK channel activity in endothelial cells is decreased, and dysfunction is associated with EDH damage [117,118]. However, in mice with chronic hypoxia and in mice with PH induced by vascular endothelial growth factor receptor antagonists, endothelial SK channel activity in small PAs did not change. Patch-clamp analysis of PA endothelial cells from control mice and PH mice revealed that after a 250 ms ramp voltage of − 100 to +50 mV was applied, the continuous addition of 1 μmol/L NS309 (IK/SK channel agonist), 1 μmol/L TRAM-34 (IK channel inhibitor) or 300 nmol/L apamin (SK channel inhibitor) was compared. There was no difference in the outward current of ECs between the control and PH groups. In addition, in the presence of 3 μmol/L free cytoplasmic Ca^2+^, the sensitivity of IK and SK channels to Ca^2+^ was similar between the control and PH groups, and the difference in channel currents between the two groups was not statistically significant, suggesting that endothelial IK and SK channel activities did not change in this PH model. Short-term acute treatment with IK/SK channel activator (SKA-31) reduced PAP in PH mice, and long-term treatment with the IK/SK channel activator reduced the collagen content and α-actin expression in PAs in PH mice, alleviating PA fibrosis. Chronic IK/SK channel activation has a beneficial effect on PA remodeling in PH [119].

### Effects of other ion channels on vascular cells and vascular fibrosis remodeling

#### Piezo1 channels in smooth muscle cells and endothelial cells and vascular fibrosis

Piezo1 is a permeable nonselective Ca^2+^ cation channel on the cell membrane that is activated by mechanical stimulation. It can serve as a key cellular mechanical sensor that converts mechanical stimulation into electrical signals [120]. One of the main functions of Piezo1 is to sense and respond to mechanical stimuli. The opening of this type channel leads to an increase in the intracellular calcium ion concentration, often triggering a variety of signaling pathways that regulate biological effects such as apoptosis, proliferation, differentiation, and migration [121]. Piezo1 is involved in the functional regulation and pathophysiological response of the cardiovascular system, and Piezo1 in smooth muscle cells is involved in hypertension-dependent arterial remodeling. Under hypertensive conditions, the opening of Piezo1 ion channels in the SMC causes thickening of blood vessel diameters and walls. Knocking down Piezo1 in SMCs inhibits this phenomenon. The opening of Piezo1 increases the Ca^2+^ concentration in the cytoplasm of VSMCs, and Ca^2+^ binds to the cross-linking enzyme aminotransferase 2 (TG2), resulting in molecular relaxation and widening and exposing the active site, thus conferring catalytic activity on TG 2 [122,123]. Huelsz et al [124] reported that TG2 activation is involved in the remodeling of small arteries in hypertensive conditions. In terms of mechanism, activated TG2 catalyzes posttranslational modifications of proteins at the Gln side chain, such as amine incorporation or cross-linking with Lys residues, due to the formation of isopeptide bonds [123]. These covalent crosslinks are used to stabilize and provide rigidity to the ECM and protect its elements from proteolytic degradation [125]. In addition, many ECM proteins act as enzyme substrates for TG2, including fibronectin, fibrinogen, collagen, and laminin [126]. Piezo 1-Ca^2+^-TG2 is closely related to the regulation of ECM remodeling, and in disease states such as hypertension, affecting one of these links may lead to arterial remodeling.

Piezo1 is also highly expressed in endothelial cells and acts as a determinant of blood vessel structure during early development [122]. Piezo1 in vascular endothelial cells responds to increased blood pressure and increased blood fluid flow during exercise. The opening of the channel causes an increase in the intracellular Ca^2+^ concentration, generating a depolarized membrane potential. The depolarized membrane potential diffuses into adjacent VSMCs via cellular gap junctions (MEGJs), activating voltage-gated Ca^2+^ channels in the VSMC, and the VSMCs depolarize, causing vasoconstriction [127,128]. The ability of the IK/SK channel inhibitors apamin and charybdotoxin to inhibit EDH (F)-mediated endothelium-dependent dilation has been widely reported [129]. Beech et al [128] reported that in WT mice, the mesenteric artery relaxation response induced by Ach can be slightly inhibited by IK/SK channel inhibitors. In the mesenteric artery of EC Piezo 1^−/−^ mice, IK/SK channel inhibitors strongly inhibited the Ach-induced diastolic response, suggesting that endothelial Piezo 1 channels can counteract EDH (F)-mediated endothelium-dependent diastole; however, the specific mechanism is still unknown. In addition, Piezo1 expressed on endothelial cells is associated with the development of vascular inflammation, which is exacerbated by the overopening of the Piezo1 channel and an inflammatory cascade activated by mechanical signals in the cells. Hong et al [130] reported that in an incomplete carotid artery ligation model, blood flow disturbance stimulated the opening of the Piezo1 pathway, resulting in a large amount of calcium inflow and triggering the release of downstream inflammatory factors, thus promoting vascular inflammation. The absence of Piezo1 in ECs effectively inhibited the vascular inflammation induced by carotid artery ligation in mice. In vitro, endothelial Piezo1 knockdown inhibited calcium influx and reduced downstream proinflammatory cytokine release. Considering the unique role of Piezo1 in smooth muscle cells and endothelial cells, Piezo1 May be a potential therapeutic target for vascular inflammatory diseases, hypertension-dependent arterial remodeling and fibrosis.

#### Upper volume-regulated anion channels (VRAC) of fibroblasts and remodeling in vascular fibrosis

The VRAC family of anion channels is composed of five LRRC8 subunits (LRRC8A-E), each encoded by its respective LRRC8 gene (LRRC8A-E). The LRRC8 subunit is assembled in a variety of configurations to produce hexameric transmembrane channel proteins, and VRAC has been shown to play a key role in cell action potential generation, cell proliferation, differentiation, and apoptosis [131,132]. Hypertension is closely related to vascular remodeling. Research on VRAC and vascular remodeling in hypertension is related. Ben et al [133] reported that the weakening effect of melatonin on fibrosis and vascular remodeling observed in animal models of hypertension and pulmonary fibrosis may be partly related to delayed fibroblast migration. In this process, melatonin restricts fibroblast overmigration and proliferation due to a dose-dependent decrease in volume-regulated anion channel (VRAC) activity mediated by protein kinase C, thus reducing fibrosis and vascular remodeling. Under the influence of hypertension, vascular smooth muscle cell proliferation and migration, endothelial cell dysfunction, inflammation and fibrosis in the cerebral basilar segment eventually lead to vascular remodeling in cerebral resistance arteries, and these change were increased expression of ClC-3 and VRAC [134]. Abnormal expression and dysfunction of VRAC in the vascular system in disease states, such as hypertension, lead to abnormal electrical activity of VRAC channels, which is related to the relationship between VRAC channel electrical activity and vascular remodeling or fibrosis. Yin et al [135] reported that the knockdown or overexpression of chloride channel 3 (ClC-3) in keratinocytes or fibroblasts affects volume-regulated anion channel Cl currents and cell differentiation. In fibroblasts overexpressing ClC-3 without exogenous TGF-β stimulation, the activity of the Cl current was significantly increased, and the level of α-SMA protein was increased. Even in the presence of TGF-β stimulation, CL-current activity and α-smooth muscle actin (α-SMA) protein levels in ClC-3 knockdown fibroblasts were reduced. VRAC and ClC-3 can play important roles in the regulation of cell function, and their respective channel mediated current and downstream signals are involved in the regulation of the transformation of fibroblasts into myofibroblasts, thus contributing to tissue and organ profibrotic actions.

#### L-type calcium ion channels in vascular smooth muscle cells (VSMCs) and vascular fibrosis remodeling

L-type Ca^2+^ channels are widely distributed in the cardiovascular system and play a key role in regulating cardiac muscle contractility [136] and vascular tension [137]. The L-type Ca^2+^ channel is composed of four distinct polypeptide subunits, of which the pore-forming subunit alpha (1) is the most important part of the channel [136]. L-type Ca^2+^ channels contain high-affinity binding domains for different chemical classes of drugs (Ca^2+^ channel antagonists; consider isradipine, verapamil, and diltiazem) within their α1 subunits. Their stereoselective, high-affinity binding blocks channel-mediated inward Ca^2+^ flow in the heart and smooth muscle, resulting in antihypertensive and antiarrhythmic effects [138]. L-type Ca^2+^ channels are closely related to many aspects of cardiovascular disease. For example, L-type Ca^2+^ channels are increased in the vascular smooth muscle cells of spontaneously hypertensive rats (SHRs) [139], and L-type Ca^2+^ channels are upregulated in the mesenteric and skeletal arteries of SHRs [140]. With respect to the relationship between L-type Ca^2+^ channels and fibrosis, Mukherjee et al [141] reported that external growth factors regulate the function of fibroblasts and are involved in the development of fibrotic disease, not only through typical signal transduction pathways but also through the propagation of periodic Ca^2+^ oscillations. Stimulation with 1 nM TGF-β1 can cause recurrent Ca^2+^ oscillation in fibroblasts. The application of 1 μM nifedipine or 1 μM verapamil (both are L-type Ca^2+^ channel blockers) significantly reduced the frequency of TGF-β1-mediated Ca^2+^ oscillation. In this process, L-type Ca^2+^ channels participate in external Ca^2+^ inflow and the propagation of Ca^2+^ oscillations. Therefore, targeting L-type Ca^2+^ channels, such as the use of L-type Ca^2+^ channel blockers, can play a role in the treatment or prevention of vascular fibrosis. This phenomenon has also been confirmed in numerous animal model experiments, including that of Teresa et al [142],who reported that daily intraperitoneal injection of nifedipine (30 mg/kg body weight) for four consecutive weeks in transgenic hypertensive TGRen2 rats reduced the proportion of type I and III glue around renal vessels and significantly improved perivascular fibrosis. Rossi et al [143] reported an increase in immature VSMCs and fibrous collagen deposition in the abdominal aortas of transgenic hypertensive TGRen2 rats. Four weeks of continuous administration of nifedipine (30 mg/kg) protected TGR from collagen deposition but had a weak effect on blood pressure. These authors analyzed the reasons for the lack of decrease in blood pressure. Since the pressure-enhancing effect of Ang II greatly exceeds the mechanism mediated by L-type Ca^2+^ channels in this model, an increase in the proportion of immature VSMCs in the TGR leads to a decrease in the proportion of L-type Ca^2+^ channel expression, thereby creating resistance to calcium channel blockade [144].

#### Smooth muscle cell ORAI1/STEM1 channels and vascular fibrosis remodeling

**Figure 2.**
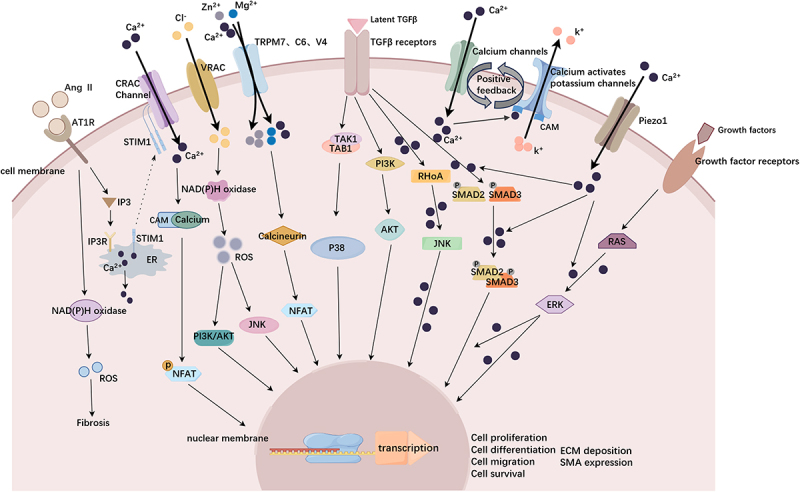
The associated ion channels and downstream signaling pathways that promote vascular fibrosis remodeling.

STIM1 and Orai1 are expressed in several cell types in the cardiovascular system, including smooth muscle cells and endothelial cells [145]. STIM molecules play important roles in store-operated Ca^2+^ entry (SOCE) through CRAC channels. Orai1 is a pore-forming component of the CRAC channel and is essential for CRAC channel activation. STIM1 is expressed on the cell surface and within the endoplasmic reticulum (ER), and inhibiting STIM1 expression prevents SOCE and eliminates the storage-dependent activation of CRAC channels. STIM1 functions as a sensor protein involved in the activation of SOCE by ER Ca^2+^ [146]. With respect to the relationship between STIM/ORAI and vascular remodeling, studies have shown that STIM1 is upregulated in the carotid arteries of injured rats with balloon injury [147]; Bisaillon et al [148] reported that the expression of the Orai1 protein was upregulated in the middle media and neointima of the carotid artery in rats with balloon injury. The administration of adenoviruses encoding short hairpin RNA (shRNA) targeting STIM1 at the site of balloon injury inhibited STIM1 upregulation, as did the formation of new intimae and the activation of the transcription factor NFAT. At the cellular level, the protein expression of STIM1 and Orai1 in synthetic VSMCs is increased, accompanied by an increase in SOCE [149]. Knockdown of STIM1 or Orai1 significantly reduces platelet-derived growth factor (PDGF)-activated SOCE, as well as VSMC proliferation and migration [150]. STIM/ORAI proteins mediate SOCE, which drives the fibroproliferative gene program during cardiovascular remodeling. STIM/ORAI-mediated SOCE drives transcriptional gene programs, including activated T nuclear factor (NFAT) transcription factors, which promote the occurrence of vascular remodeling in vitro and in animal models of restenosis and hypertension [151]. Considering the role of the SOCE pathway and STIM1/Orai1 proteins in driving smooth muscle proliferation and migration, these channel proteins may be used as therapeutic targets for vascular remodeling. As depicted in Figure 2: The associated ion channels and downstream signaling pathways that promote vascular fibrosis remodeling.

## Conclusions

Vascular fibrosis is not regarded as a specific disease. In fact the occurrence and development of many vascular diseases are accompanied by pathologically abnormal vascular fibrosis. The extent and rate the remodeling of vascular fibrosis is regulated by chronic inflammatory responses caused by various stimuli, which eventually leads to excessive deposition of collagen and changes in the composition of the extracellular matrix (ECM). Inhibition of the persistent fibrotic response involves the regulation of the activation and phenotypic transformation of smooth muscle cells, fibroblasts and endothelial cells by specific cytokines, growth factors, stromal cell proteins, integrins, mechanosensitive signaling pathways and ion channels to achieve the therapeutic purpose of alleviating fibrosis. In conclusion, the complex mechanisms involved in arterial fibrosis make anti-fibrosis strategies challenging. From the perspective of ion channels on vascular wall cells, exploring their signal transduction processes and the downstream signaling pathways they activate may help us find more appropriate strategies to combat the pathological abnormality of vascular fiber remodeling.

## Data Availability

Data sharing is not applicable to this article, as no new data were created or analyzed in this study.
